# From polygenic risk to digital twins: the future of personalised cardiovascular medicine

**DOI:** 10.3389/fcvm.2026.1735094

**Published:** 2026-02-09

**Authors:** Ibrahim Antoun, Alkassem Alkhayer, Ahmed Abdelrazik, Mahmoud Eldesouky, Kaung Myat Thu, Mokhtar Ibrahim, Harshil Dhutia, Riyaz Somani, G. Andre Ng

**Affiliations:** 1 Department of Cardiology, University Hospitals of Leicester NHS Trust, Glenfield Hospital, Leicester, United Kingdom; 2 Department of Cardiovascular Sciences, Clinical Science Wing, University of Leicester, Glenfield Hospital, Leicester, United Kingdom; 3 Department of Cardiology, Guys and St Thomas Hospital, London, United Kingdom; 4 National Institute for Health Research Leicester Research Biomedical Centre, Leicester, United Kingdom; 5 Leicester British Heart Foundation Centre of Research Excellence, Glenfield Hospital, Leicester, United Kingdom

**Keywords:** artificial intelligence, digital twin technology, multi-omics biomarkers, polygenic risk scores, precision cardiology

## Abstract

Cardiovascular disease (CVD) remains the leading cause of morbidity and mortality worldwide. Traditional risk assessment and treatment approaches often follow generalised strategies that inadequately capture individual variability in disease susceptibility, progression, and therapeutic response. Precision cardiology seeks to overcome these limitations by leveraging genomic, molecular, and computational innovations to enable more individualised care. Advances in polygenic risk scores have improved our ability to stratify cardiovascular risk at a population level, though challenges remain in ensuring clinical utility across diverse populations. Integrating multi-omics platforms, including transcriptomics, proteomics, and metabolomics, offers a more comprehensive understanding of CVD pathophysiology and potential diagnostic or prognostic biomarkers. Pharmacogenomic insights increasingly guide the selection and dosing of cardiovascular therapies such as statins and antiplatelets, supporting the shift toward personalised pharmacologic strategies. Applying artificial intelligence and machine learning to cardiovascular imaging, electronic health records, and wearable data enables more accurate, scalable predictive models. Emerging technologies, including CRISPR-based gene editing, single-cell sequencing, and digital twin modelling, further expand the frontiers of personalised cardiovascular medicine. However, real-world implementation remains limited by regulatory uncertainty, data integration challenges, cost, and concerns about equity and access. This review synthesises advances across genomic, omics, digital, and therapeutic domains in cardiovascular precision medicine, discusses key translational gaps, and highlights ethical and implementation challenges. We emphasise the need for multidisciplinary collaboration, robust validation frameworks, and equitable infrastructure to ensure these innovations lead to meaningful clinical impact. Personalised cardiology is poised to redefine prevention, diagnosis, and treatment paradigms as the field matures, moving from reactive care to proactive, patient-specific strategies.

## Introduction

Cardiovascular diseases (CVDs) pose a tremendous public health burden, and their management is challenging in the developing world (1–5). Conventional approaches to CVD prevention and management rely on treating established risk factors (such as hypertension, hyperlipidaemia, and diabetes) and applying population-derived risk algorithms. While these strategies have reduced CVD mortality over the past decades, many individuals classified as “low-risk” still develop disease, and treatment responses vary widely. This underscores a critical need for more nuanced risk stratification and individualised therapy, the central tenets of personalised (or precision) cardiovascular medicine. Personalised medicine seeks to integrate an individual's unique genetic, molecular, and environmental profile to predict disease and tailor interventions more accurately (6).

Recent technological and scientific advances have created new opportunities to personalise cardiovascular care. On the genetic front, large genome-wide association studies have identified numerous DNA variants that influence CVD risk, enabling the development of polygenic risk scores that aggregate these variants into a single risk estimate for an individual (7). Similarly, high-throughput profiling of the “omes”—the genome, epigenome, transcriptome, proteome, metabolome, and microbiome—provides a multidimensional molecular picture of patients. Such *multi-omics* approaches can reveal novel biomarkers and mechanistic pathways underlying CVD that are not apparent from traditional factors (8). In parallel, the field of *pharmacogenomics* has matured, identifying genetic variants that modulate responses to cardiovascular drugs like clopidogrel, warfarin, and statins (9). This knowledge is beginning to inform therapy selection to maximise efficacy and minimise adverse effects for individual patients.

Another revolution in personalised care comes from data science. Artificial intelligence (AI) and machine learning (ML) techniques excel at detecting complex patterns in large datasets. In cardiovascular medicine, AI/ML are being applied to diverse data sources, including imaging, electrocardiography, electronic health records, and wearable sensors, to enhance diagnostic accuracy, risk prediction, and clinical decision-making (10–13). At the same time, breakthroughs in *gene editing* (notably CRISPR-Cas9 technology) raise the possibility of directly correcting or mitigating pathogenic genetic factors, as exemplified by initial *in vivo* gene-editing trials for lipid disorders (14).

This narrative review explores the evolving landscape of personalised cardiovascular medicine, spanning polygenic risk prediction, multi-omics biomarkers, pharmacogenomics, artificial intelligence, gene editing, and single-cell genomics. Rather than treating these domains as independent innovations, we conceptualise them as upstream components of an emerging integrative paradigm centred on data fusion, mechanistic modelling, and individualised decision support. In this framework, digital twin technologies represent a natural convergence point, synthesising molecular, clinical, imaging, and longitudinal sensor data into patient-specific computational models that can forecast disease trajectories and simulate therapeutic scenarios. Accordingly, this review places particular emphasis on the translational logic linking molecular stratification to system-level modelling, with the aim of illustrating how disparate precision tools may ultimately coalesce into unified, clinically actionable platforms. We also discuss the ethical, regulatory, and implementation challenges that must be addressed to ensure that these advances translate into equitable and meaningful patient benefits. In this review, we use the terms “personalised medicine” and “precision medicine” interchangeably to refer to the tailoring of prevention, diagnosis, and treatment based on individual-level biological, clinical, and contextual data. While “precision” often emphasises molecular stratification and data-driven modelling, and “personalised” emphasises patient-centred tailoring, both terms describe a shared paradigm shift away from population-average approaches toward individualised decision-making.

## Polygenic risk scores: genetic risk prediction in cardiovascular disease

Genome-wide association studies over the past 15+ years have identified hundreds of genetic loci associated with coronary artery disease, atrial fibrillation, stroke, and other cardiovascular conditions (15). Individually, common variants usually confer modest risk increments, but collectively, a person's burden of risk alleles can substantially influence disease susceptibility. *Polygenic risk scores (PRS)* quantify this inherited risk by summing the effects of numerous risk-associated single-nucleotide polymorphisms (SNPs) into a single score for each individual. PRS are typically constructed from large discovery cohorts and can encompass anywhere from dozens to millions of variants, weighted by their effect sizes from GWAS. In essence, a PRS is a personalised genetic risk factor present from birth.

### Development of PRS

Initial PRS models for coronary artery disease (CAD) and other CVDs emerged in the late 2010s. For example, a 2018 study by Khera et al. combined >6 million variants into a PRS for CAD that identified individuals in the top 5% of genetic risk with a three-fold higher odds of coronary disease (16). Such early scores demonstrated that a person's polygenic susceptibility could be as powerful as having a single-gene disorder or a major clinical risk factor. However, a major limitation quickly became evident: most PRS were derived from European-ancestry GWAS and showed reduced predictive accuracy in non-European populations (17). This *lack of portability* raised concerns about equity, since the utility of PRS would be limited in underrepresented groups. In response, researchers have developed multi-ancestry PRS using diverse cohorts. A landmark 2023 effort integrated data from over 1 million individuals across multiple ancestries (including European, African, South Asian, and Hispanic) to produce an improved PRS for coronary artery disease (18). This score, termed GPS^Mult, leveraged not only genomic variants for CAD but also genetic signals from related traits such as blood pressure and BMI to improve performance. The multi-ancestry PRS achieved superior accuracy in predicting coronary risk across diverse populations compared to prior European-only scores. Notably, it identified a subset (∼3%) of individuals with polygenic risk scores comparable to those of patients with established disease, underscoring the potential of genetics to flag high-risk individuals early in life (18). As PRS methods continue to improve, through larger GWAS, more sophisticated statistical algorithms, and the inclusion of non-European data, the ceiling for predictive power has not yet been reached. PRS for other CVDs are likewise being refined, often demonstrating modest but significant predictive value when added to clinical risk factors (19).

### Clinical utility

The promise of PRS is the ability to stratify individuals by inherited risk *long before* disease manifests. Unlike most traditional risk factors, a person's PRS is fixed at conception and thus could enable early-life risk prediction and prevention. For instance, an individual found to have a top-percentile PRS for coronary disease (conferring, say, a 2–4 fold risk increase) might benefit from aggressive preventive measures decades earlier than standard guidelines would indicate. Modelling studies in the UK Biobank have shown that adding a PRS to conventional risk factors modestly improves risk discrimination (C-index increase ∼0.01) and net reclassification of individuals at risk for coronary events (19). The improvement in predictive power, while incremental at the population level, could be clinically meaningful for certain patients, particularly those on the cusp of treatment thresholds. One analysis estimated that universal PRS screening of UK adults aged 40–75 could prevent one additional cardiovascular event per ∼5,750 people screened under current statin guidelines, whereas targeting PRS testing to those at intermediate 5%–10% 10-year risk could prevent one event per ∼340 screened (19). Such a targeted strategy might avert ∼7% more events than using clinical risk factors alone. These data suggest PRS could serve as a “risk-enhancer,” much like a strong family history, coronary calcium score, or high-sensitivity C-reactive protein, to refine preventive therapy decisions. In fact, recent consensus statements have proposed that an extreme PRS (top 1%–5%) be considered as an indicative risk factor to justify earlier intervention (20).

Emerging clinical studies are now evaluating the real-world impact of disclosing PRS and intervening on the basis of that disclosure. For example, the ongoing INNOPREV trial is a randomised study testing whether providing individuals with their CVD genetic risk (PRS) and digital health tools can motivate lifestyle change and improve risk profiles beyond standard care (21). Early evidence also hints at psychological and behavioural effects of knowing one's genetic risk. A recent meta-analysis found that individuals informed of a high genetic risk for disease were more likely to adopt healthy behaviours (such as improved diet and exercise) than controls (22). However, results have varied and long-term behaviour change remains uncertain. Overall, PRS implementation in clinical care is still nascent. Some pioneering healthcare systems have begun pilot programs to calculate PRS for patients (in specialised preventive cardiology clinics), but routine use is not yet widespread.

### Limitations

Several challenges must be addressed before PRS fully integrates into cardiovascular practice. First, *ancestry bias* in PRS construction has raised equity concerns; scores derived from European-centric data perform worse in African, Asian, or Hispanic populations. Concerted efforts, such as the NHLBI PRIMED Consortium, aim to diversify genomic datasets and improve PRS performance across ancestries (23). The 2023 multi-ancestry CAD score is a significant step forward, but continued inclusion of underrepresented groups in genetic research is critical to avoid widening health disparities (18). Second, the *magnitude of risk reduction* achievable through action on PRS information remains to be demonstrated. Unlike monogenic mutations (LDL receptor mutations in familial hypercholesterolemia), which clearly indicate specific interventions, a high polygenic score may warrant intensification of general prevention (earlier statin initiation, lifestyle changes), whose marginal benefit requires quantification. Third, the *clinical interpretation* and communication of PRS present practical challenges. There is no universal standard for what constitutes “high” genetic risk or how to convey this probabilistic information to patients without causing confusion or fatalism. Fourth, implementing PRS testing at scale raises logistical and economic questions; it requires DNA genotyping (or sequencing) infrastructure and strategies for selecting whom and when to test (e.g., newborn screening, young adults, or only those with intermediate risk by other measures). Cost-effectiveness analyses are only beginning to emerge; these will inform health policy on PRS screening. Finally, there are ethical and legal considerations.

### Future perspectives

Looking ahead, polygenic risk assessment is likely to become more accurate, comprehensive, and widely available. Continuous improvements in PRS construction (integrating ever-larger GWAS, functional annotations, and ML to weight variants) are expected to boost predictive performance. The concept of the “integrated risk score” may arise, combining PRS with clinical and possibly multi-omic markers to yield a composite personalised risk. Another promising avenue is using PRS not only for risk prediction but also to guide therapy, for instance, identifying individuals who might particularly benefit from certain drug classes. Early examples include using an LPA gene score to select patients for lipoprotein(a)-lowering treatments, or a PRS for diabetes to intensify lifestyle counselling.

Additionally, as PRS research expands to include more traits, we may develop scores to predict drug efficacy or side effects (polygenic *pharmacogenetic* scores), thereby further personalising treatment choices. Ultimately, polygenic risk scoring represents a paradigm shift from reactive treatment to proactive prevention in cardiology.

## Beyond common variants: rare variants, somatic mutations, and CHIP

While most contemporary PRS models are derived from common germline variants (24), emerging evidence indicates that rare variants and acquired somatic mutations also contribute meaningfully to cardiovascular risk (25). Clonal hematopoiesis of indeterminate potential, defined by the age-related expansion of hematopoietic clones carrying somatic mutations in genes such as DNMT3A, TET2, ASXL1, and JAK2, has been associated with increased risks of coronary artery disease, heart failure, stroke, and mortality (26). These effects appear to be mediated, in part, by pro-inflammatory signalling and altered immune cell function, rather than by traditional lipid or blood pressure pathways. Importantly, CHIP represents a dynamic, tissue-specific form of genetic risk that evolves over time, challenging the static assumptions underlying conventional PRS.

Attempts are now underway to integrate rare pathogenic variants and somatic mutations with polygenic background risk into unified genetic risk frameworks. Such hybrid models aim to capture the full allelic spectrum of cardiovascular susceptibility, ranging from rare high-impact variants to common low-effect polymorphisms. However, this integration poses substantial challenges (27). Rare variants often exhibit population-specific effects and incomplete penetrance, while somatic variants require deep sequencing for detection and may vary across tissues and over time. Moreover, the effect sizes of CHIP-associated mutations appear context-dependent and may interact with environmental exposures and ageing.

From a clinical perspective, incorporating rare and somatic variants into PRS raises unresolved questions about screening thresholds, longitudinal monitoring, and intervention strategies. Unlike germline PRS, which can be measured once, CHIP burden may require repeated assessment. There is also limited evidence on whether targeted interventions in CHIP-positive individuals reduce cardiovascular risk. As such, next-generation risk models will likely need to move beyond static polygenic scores toward temporally adaptive, multi-layered genomic profiles that integrate germline, rare, and somatic variation. This evolution aligns closely with the broader vision of precision cardiology, in which genetic risk is treated as dynamic, contextual, and modifiable rather than fixed.

## Multi-omics biomarkers for diagnosis and risk stratification

While genetics provides a foundational risk baseline, dynamic molecular changes captured by *omics* technologies offer another layer of personalisation. Multi-omics refers to the integrated analysis of multiple biological “omes,” such as the genome, epigenome, transcriptome (RNA expression), proteome (protein levels), metabolome (metabolites and lipids), and microbiome. Each omics layer can yield biomarkers, measurable indicators of physiological or pathological processes, that improve understanding of disease mechanisms and refine risk assessment or diagnosis beyond classical clinical markers (28).

### Challenges of tissue specificity and the limits of circulating biomarkers

A fundamental challenge in cardiovascular omics is the limited accessibility of human cardiac tissue (29). Unlike oncology, where tumour biopsies are often available, routine myocardial sampling is invasive, risky, and rarely justified outside of select clinical contexts such as transplant rejection, infiltrative cardiomyopathies, or unexplained myocarditis. As a result, most large-scale omics studies rely on plasma-based biomarkers, which offer convenience and scalability but provide only an indirect window into myocardial biology. Circulating proteins, metabolites, and RNA species often reflect systemic inflammation, hepatic metabolism, renal clearance, or immune activation rather than cardiac-specific processes, complicating mechanistic interpretation (30).

Recent efforts have attempted to infer tissue of origin using integrative approaches, including linking circulating markers to tissue-specific expression quantitative trait loci, single-cell atlases, and spatial transcriptomic maps of the human heart (29). However, these methods remain probabilistic and cannot fully substitute for direct tissue-level profiling. Moreover, many cardiovascular phenotypes, such as myocardial fibrosis, microvascular dysfunction, or arrhythmogenic substrate formation, are spatially heterogeneous and may not be captured by bulk plasma signals. Future progress will depend on combining plasma omics with higher-resolution modalities, including cardiac imaging, electroanatomic mapping, and tissue-informed molecular atlases (31). Advances in minimally invasive sampling, cell-free RNA profiling, exosome analysis, and spatial transcriptomics may help bridge this gap. In precision cardiology, it will be essential to recognise that circulating biomarkers are proxies rather than direct measures of myocardial pathology, and that multimodal integration will be required to infer underlying tissue-level mechanisms and to guide individualised therapy (32).

### Novel biomarker discovery

Omics approaches have already identified numerous biomarkers with potential clinical relevance in CVD. For example, metabolomics (using technologies like NMR and mass spectrometry) has uncovered metabolic signatures associated with cardiovascular risk. Elevated plasma levels of certain metabolites, such as trimethylamine N-oxide (TMAO, a gut microbiome-derived molecule), branched-chain amino acids, or specific lipid species (e.g., ceramide subclasses), have been linked to an increased incidence of CAD and adverse outcomes (33). In some cases, these markers provide predictive information beyond traditional risk factors, indicating they capture novel aspects of pathology. Indeed, a *ceramide risk score* assay has been developed and shown to stratify risk of major adverse cardiovascular events, prompting incorporation of ceramide testing in a few preventive cardiology clinics (34).

Proteomics has emerged as one of the most clinically translatable omics platforms in cardiovascular medicine, owing to the relative stability of circulating proteins, the availability of high-throughput assays, and the direct mechanistic relevance of proteins as drug targets. Recent large-scale proteomic studies using aptamer-based and mass spectrometry platforms have profiled thousands of circulating proteins in population cohorts and disease-specific samples (35), revealing distinct protein signatures across a wide range of cardiovascular conditions. For example, proteomic risk models have been developed for incident coronary artery disease, heart failure, atrial fibrillation, and cardiovascular mortality, often outperforming traditional risk scores when combined with clinical variables (36, 37).

Beyond risk prediction, proteomics enables mechanistic endophenotyping. In heart failure, plasma proteomic profiling has identified molecular subtypes characterised by inflammatory, fibrotic, metabolic, or angiogenic signatures, each associated with distinct prognoses and therapeutic responses (37). In atherosclerosis, proteomic analyses of plaque tissue and plasma have revealed pathways related to immune activation, lipid handling, and extracellular matrix remodelling, offering insight into plaque vulnerability rather than luminal stenosis alone (38). Similarly, in AF, proteomic markers of atrial remodelling, fibrosis, and oxidative stress have been linked to recurrence after ablation and progression to persistent disease (39).

Proteomics has also become a powerful tool for therapeutic target discovery and validation. Integration of quantitative trait loci (QTLs) with proteomic data has enabled causal prioritisation of drug targets and identification of repurposing opportunities. For instance, genetically anchored proteomic studies have uncovered novel mediators of myocardial remodelling, vascular inflammation, and thrombosis that are now being explored in early-phase drug development. Importantly, because most drugs act on proteins, proteomic platforms provide a more direct bridge between molecular discovery and pharmacological translation than transcriptomic or epigenomic approaches (40).

However, important challenges remain. Proteomic measurements are influenced by comorbidities, medications, and acute physiological states, complicating interpretation. Platform-specific variability and limited standardisation across assays remain barriers to clinical deployment. Moreover, many proteomic signatures remain unlinked to specific therapeutic actions, limiting their immediate utility. Future work will need to focus on harmonising assays, validating disease-specific protein panels, and embedding proteomic signatures into decision-support frameworks. In the context of precision cardiology, proteomics is likely to serve as a key intermediate layer that connects genetic predisposition with dynamic disease biology and actionable interventions.

### Plasma transcriptomics and extracellular vesicles as surrogates of tissue-level biology

To address the limited availability of cardiac tissue, recent studies have explored plasma transcriptomics, particularly by analysing extracellular vesicles (EVs), including exosomes and microvesicles (41). These vesicles are actively secreted by cells and contain RNA species, including mRNA, microRNA, and long non-coding RNA, that can reflect the transcriptional state of their tissue of origin. Unlike freely circulating nucleic acids, EV-encapsulated RNA is relatively stable and protected from enzymatic degradation, making it an attractive substrate for longitudinal molecular profiling.

In cardiovascular disease, EV-based transcriptomic profiling has been investigated in conditions such as myocardial infarction, heart failure, AF, and inflammatory cardiomyopathies. Emerging evidence suggests that EV cargo may encode information about cardiomyocyte stress, endothelial dysfunction, immune activation, and fibrotic remodelling, offering a more tissue-proximal signal than conventional plasma proteomics or metabolomics alone. These approaches may enable non-invasive tracking of myocardial remodelling, arrhythmogenic substrate evolution, or response to therapy over time (42, 43).

However, several challenges remain. EV populations are heterogeneous, isolation methods lack standardisation, and tissue-of-origin assignment is often indirect. Moreover, the biological meaning of many circulating RNA species remains incompletely understood. Large-scale validation studies are still scarce, and clinical actionability has yet to be established (44). Nonetheless, EV-based plasma transcriptomics represents a promising intermediate between direct tissue profiling and systemic biomarkers. In the context of personalised cardiovascular medicine, it may help bridge the gap between molecular mechanisms and scalable clinical testing.

### Clinical translation and use cases

The translational value of multi-omics biomarkers lies in their ability to *augment clinical decision-making* at various stages. In prevention, a panel of omics-based biomarkers could improve risk stratification by identifying high-risk individuals whom standard risk calculators might otherwise miss. For example, adding an aggregate metabolomic risk score to a Framingham Risk Score model has been shown to reclassify a significant proportion of individuals into higher- or lower-risk categories, potentially altering management (8). In diagnosis, multi-omics can aid in earlier detection or more accurate differentiation of cardiovascular conditions. One notable area is *precision medicine in cardiomyopathies*: genetic and transcriptomic analyses of patients with cardiomyopathies have helped distinguish hypertrophic vs. dilated cardiomyopathies of different etiologies (e.g., sarcomeric mutations, amyloidosis), enabling tailored treatments (45, 46). Similarly, the integration of proteomic and metabolomic markers is being tested to improve diagnostic accuracy for obstructive CAD and acute coronary syndromes, potentially reducing reliance on invasive testing. Another emerging application is in *predicting treatment response*. For instance, in heart failure, researchers have identified plasma protein signatures that predict which patients are more likely to respond to therapies like beta-blockers or cardiac resynchronisation therapy. This could lead to omics-guided therapy selection in the future.

It is worth noting that some multi-omics findings have already begun to influence clinical practice in subtle ways. As noted, high-sensitivity troponin (a highly sensitive proteomic marker of cardiomyocyte injury) is now used not only for acute diagnosis but also to assess chronic risk in asymptomatic populations. Elevated baseline troponin T or I levels correlate with higher long-term CVD risk, even below the threshold of clinical myocardial infarction. Likewise, Lp(a), an atherogenic lipoprotein with a strong genetic determination (and sometimes considered part of the “genomic” risk profile) (47), is now recommended for one-time measurement in adults to identify those with very high inherited levels (48). These examples illustrate a broader trend: *omics-informed biomarkers are gradually being incorporated into guidelines* as evidence accumulates [for example, European guidelines acknowledge the value of Lp(a) and high-sensitivity troponin as risk refiners] (48).

### From association to causation: Mendelian randomisation and target prioritisation

While multi-omics approaches can identify numerous candidate biomarkers and molecular pathways, distinguishing causal mediators from correlates remains a major translational challenge. Mendelian randomisation (MR) provides a powerful framework for causal inference by using germline genetic variants as instrumental variables to test whether an exposure or biomarker lies on the causal pathway to disease. In cardiovascular research, MR has been instrumental in validating therapeutic targets (49, 50), including LDL cholesterol, PCSK9, and IL-6 signalling, while deprioritising others that showed strong observational associations but lacked causal effects. This approach reduces confounding and reverse causation, thereby increasing confidence that modulating a given biomarker will alter disease risk.

More recently, MR has been extended to high-dimensional proteomic, metabolomic, and transcriptomic datasets, enabling systematic causal screening of hundreds of molecular traits. These multi-omic MR pipelines help prioritise drug targets, inform repurposing strategies, and guide early-phase drug development. However, important limitations remain. Horizontal pleiotropy, weak instruments, and ancestry-specific effects can bias causal estimates. In addition, MR typically reflects lifelong genetic perturbations, which may not fully recapitulate the effects of short-term pharmacological interventions. Tissue specificity and temporal dynamics further complicate interpretation, particularly for immune- and inflammation-related pathways.

Despite these challenges, causal inference methods are increasingly viewed as a necessary complement to association-based discovery (51). Integrating MR with longitudinal multi-omics, single-cell data, and functional validation offers a more rigorous pipeline for moving from biomarker identification to mechanism-informed therapy. In the context of personalised cardiovascular medicine, such frameworks will be essential to ensure that molecular stratification translates into interventions that modify disease trajectories rather than merely refine risk prediction.

### Limitations

Despite the excitement, translating multi-omics discoveries into routine clinical tools remains a significant challenge. A primary challenge is the *complexity and volume of data*. Multi-omics studies generate high-dimensional datasets that require sophisticated bioinformatics to distil into clinically relevant information. Distinguishing true signal from noise is difficult, given issues of multiple comparisons and correlations among markers. There is a risk of false positives: proposed biomarkers that initially show association but fail to replicate or prove causality. Thus, robust validation in independent cohorts, preferably in interventional studies, is needed before adopting new biomarkers. Another challenge is *standardisation*: unlike measuring a single analyte such as cholesterol, multi-omics assays can vary across platforms and analysis pipelines, and there are not yet universally accepted cut-offs or units for many biomarkers. Ensuring reproducibility and harmonisation of assays (across different labs or vendors) is essential for clinical deployment (8).

Cost and practicality are also non-trivial issues. Comprehensive multi-omics profiling (whole-genome sequencing plus broad proteomic/metabolomic panels) remains expensive and resource-intensive. Implementing these in clinical laboratories may require new infrastructure and expertise (e.g., the installation of mass spectrometers in hospital laboratories or the outsourcing of tests to specialised facilities). Turnaround time must also be compatible with clinical needs. Moreover, many omics-based biomarkers lack clear therapeutic actionability; knowing that a patient has a specific proteomic risk signature may not yet translate into an approved intervention, thereby limiting clinical utility. There is also the question of *regulatory approval*: multi-analyte biomarker tests typically must undergo regulatory pathways that demonstrate reliability and improved clinical outcomes. Only a few multi-marker tests in cardiology (such as multi-protein risk scores for heart failure hospitalisation) have reached the market to date, often with only niche uptake.

### Future perspectives

The field of cardiovascular biomarker discovery is moving toward ever more *integrative* models. Rather than relying on single biomarkers, we will likely see composite scores or algorithms that integrate data types (genomic, proteomic, clinical, imaging) for a holistic risk assessment. ML will play a role in integrating multi-omics data; in fact, researchers are already using ML to handle high-dimensional omics inputs and identify predictive features (52). In the next decade, it is conceivable that a patient might undergo a comprehensive “omics” screening (perhaps through a single blood draw that undergoes multiplexed analysis), yielding a personalised risk dashboard: genetic risk score, key protein biomarkers, metabolite levels, each mapped to intervention options.

Precision medicine initiatives are also exploring multi-omics to guide therapy beyond risk prediction. For instance, in CAD, if certain inflammatory biomarkers are elevated (as detected by proteomic profiling), the patient may benefit from an anti-inflammatory therapy in addition to standard care. If metabolomics indicates high TMAO (suggesting adverse gut microbiome activity), dietary modifications or microbiome-targeted therapies might be recommended (53). Thus, multi-omics could stratify patients for emerging treatments, moving toward *mechanism-guided therapy*. Additionally, longitudinal multi-omics, which track how a patient's molecular profile changes over time or in response to treatment, could enable dynamic monitoring of disease activity (akin to a molecular CT scan). This is particularly relevant in conditions such as heart failure or atherosclerosis, where molecular markers may reflect improvement or progression earlier than clinical endpoints.

## Pharmacogenomics: optimising therapy through genetic insights

Inter-individual variability in drug response is a well-recognised phenomenon in cardiovascular therapeutics. Some patients experience superior benefits or, conversely, serious adverse effects from a given medication, while others do not. *Pharmacogenomics* (or pharmacogenetics) seeks to explain a portion of this variability through genetic differences, with the ultimate goal of guiding medication selection and dosing for each patient (9). Cardiovascular medicine has been at the forefront of pharmacogenomic discovery, with several landmark examples now informing clinical practice.

### Key pharmacogenomic findings in cardiology

**Figure 1:**
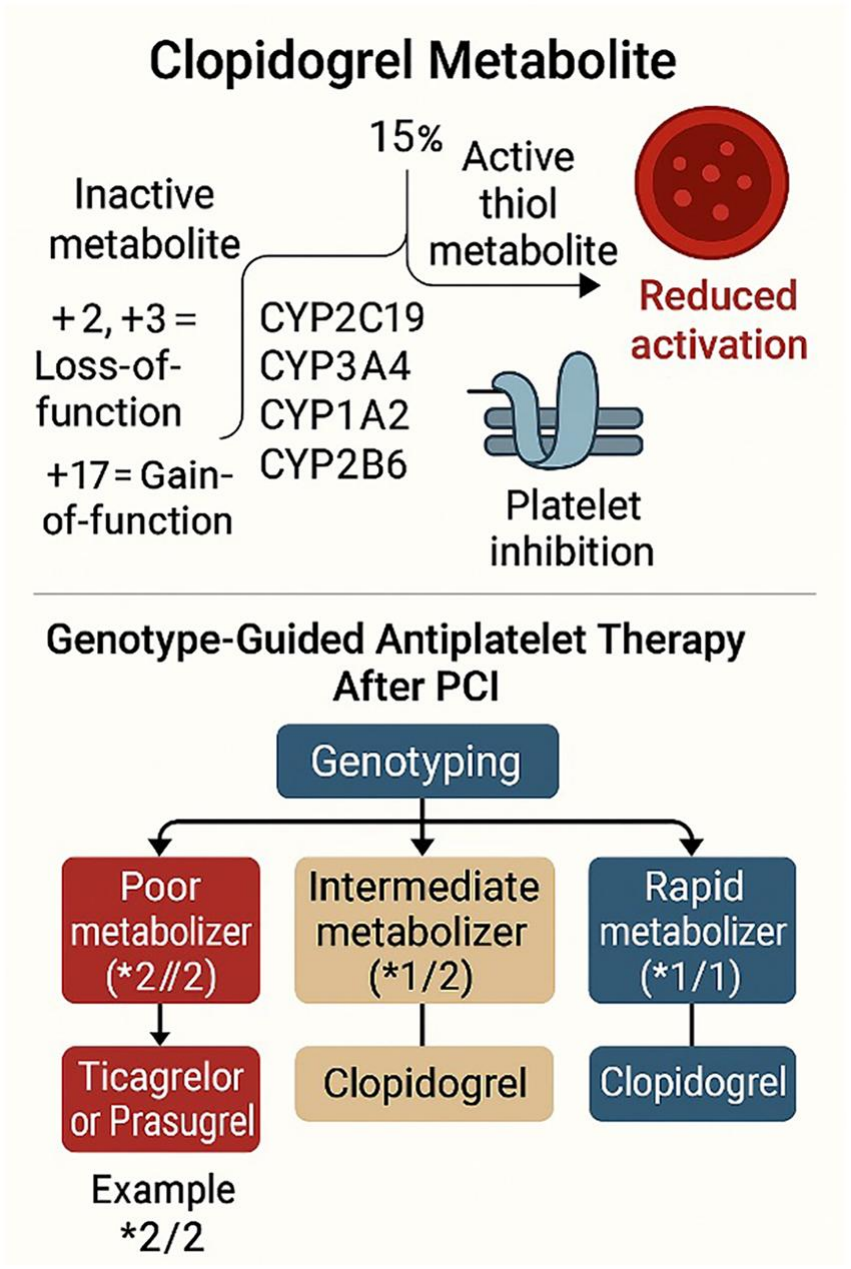
Genetic determinants of antiplatelet therapy response in precision cardiovascular medicine. Clopidogrel requires hepatic bioactivation via cytochrome P450 enzymes to form its active metabolite. Loss-of-function alleles (e.g., *2, *3) impair this process, reducing platelet inhibition and increasing risk of stent thrombosis and ischemic events. Genotype-guided therapy after PCI stratifies patients into poor metabolizers (treated with ticagrelor or prasugrel), intermediate metabolizers (treated with clopidogrel with clinical judgment), and rapid metabolizers (treated with clopidogrel). PCI, percutaneous coronary intervention.

The most widely cited case is *clopidogrel*, an antiplatelet pro-drug used to prevent thrombosis after myocardial infarction or stenting. Clopidogrel's activation in the liver is primarily mediated by the enzyme CYP2C19 (54). It was discovered that common loss-of-function variants in the CYP2C19 gene (*CYP2C19 2* allele and others) impair clopidogrel metabolism (Figure 1), leading to reduced active drug levels and higher on-treatment platelet reactivity (54, 55). Patients carrying two loss-of-function alleles (“poor metabolizers”) have significantly higher rates of stent thrombosis and recurrent cardiovascular events when treated with standard clopidogrel (9, 55). This knowledge has spurred genotype-guided antiplatelet therapy: patients with CYP2C19 loss-of-function alleles can be prescribed alternative P2Y12 inhibitors that are not dependent on that pathway (e.g., prasugrel or ticagrelor) to achieve effective platelet inhibition. Clinical trials like TAILOR-PCI have examined this approach. At the same time, results have been mixed in terms of primary outcomes (56), subgroup analyses, and meta-analyses suggest genotype-guided therapy may reduce adverse events in high-risk patients (55). Importantly, the FDA added a boxed warning to clopidogrel's label regarding reduced effectiveness in CYP2C19 poor metabolizers, and expert guidelines provide recommendations on the use of genotype information for antiplatelet selection.

Another well-established example is *warfarin*, a vitamin K antagonist anticoagulant with a narrow therapeutic index. Warfarin dosing is notoriously variable between individuals. Variants in CYP2C9 (affecting warfarin metabolism) and VKORC1 (the drug's target enzyme) explain a substantial portion of dose variability. Carriers of CYP2C9 *2* or *3* alleles metabolise warfarin more slowly, requiring lower maintenance doses, while VKORC1 promoter variants that decrease enzyme expression also lower dose requirements (57). Algorithms that incorporate a patient's genotype at these genes, along with clinical factors, can more accurately predict the optimal warfarin dose than clinical factors alone. Several randomised trials (EU-PACT, COAG) tested genotype-guided warfarin dosing, with EU-PACT showing improved time in therapeutic range and fewer out-of-range INR values (58, 59). However, the clinical impact on bleeding or thrombosis outcomes was less clear. With the rise of direct oral anticoagulants (DOACs) that do not require such dosing adjustments, warfarin pharmacogenetics did not become routine in all settings. Nonetheless, it remains a prime example of genomic precision dosing, and some centres do utilise genetic dosing algorithms for warfarin, especially for patients in whom warfarin is still the drug of choice.

For *statin* therapy, pharmacogenomics has informed risk mitigation of side effects. A common variant in SLCO1B1 (which encodes a hepatic drug transporter) is associated with impaired hepatic statin uptake and higher plasma statin concentrations. This *SLCO1B1* c.521T > C variant (*SLCO1B1 5* allele) markedly increases the risk of statin-induced myopathy, particularly with higher-dose simvastatin (60–62). The FDA also updated simvastatin labelling to caution on myopathy risk in genetically predisposed individuals. Many clinical pharmacogenetic programs now test for SLCO1B1 variants as part of a preemptive panel, given the widespread use of statins. By identifying at-risk patients, clinicians can personalise statin therapy, for example, starting with lower doses, choosing a statin with a different metabolism (pravastatin, rosuvastatin), or emphasising non-statin lipid-lowering options if necessary.

Additional pharmacogenomic associations in cardiology continue to emerge. Variants in *ADRB1* and other adrenergic pathway genes have been studied for their impact on β-blocker response in heart failure (63). One polymorphism (*ADRB1* Arg389Gly) was associated with differential improvement in left ventricular function on bucindolol (a beta-blocker) in a genotype-directed trial (64), although that drug is not widely used clinically. In African American patients with heart failure, genotypes in nitric oxide signalling pathways were initially thought to predict response to hydralazine-isosorbide dinitrate therapy. Still, subsequent analyses did not confirm a strong effect, so race-based empirical use remains the practice. Beyond cardiovascular drugs *per se*, pharmacogenetics also influences the use of frequently prescribed medications in patients with cardiovascular disease. For instance, genetic variations in CYP2D6 influence response to certain antiarrhythmics and beta-blockers, and genetic polymorphisms affect dosing of immunosuppressants in transplant cardiology (65).

Traditional pharmacogenomics has largely focused on single gene-drug pairs, such as CYP2C19 and clopidogrel or SLCO1B1 and statin intolerance (66). While clinically useful, these models capture only a portion of the genetic architecture underlying drug response. Increasingly, drug efficacy and toxicity are being recognised as complex, polygenic traits influenced by multiple variants across metabolic enzymes, transporters, receptors, downstream signalling pathways, and immune modulators. This has led to the emergence of polygenic pharmacogenomics, in which composite genetic scores are used to more comprehensively predict interindividual variability in drug response.

Early studies suggest that polygenic models may outperform single-variant approaches in predicting treatment response, adverse drug reactions, and optimal dosing across multiple drug classes (67). In cardiovascular medicine, this paradigm may be particularly relevant for statins, beta-blockers, anticoagulants, and antiarrhythmics, in which response heterogeneity persists despite guideline-directed therapy. Polygenic predictors may also help identify individuals who derive marginal benefit from certain therapies, supporting de-escalation or alternative strategies.

However, major challenges remain. Polygenic pharmacogenomic scores require large, well-phenotyped cohorts with detailed drug exposure and outcome data, which are currently limited. Effect sizes are often modest, raising questions about clinical actionability (68). Moreover, these models must be robust across ancestries and clinical contexts, and their integration into prescribing workflows will require careful validation, clinician education, and the development of decision-support tools.

In the longer term, polygenic pharmacogenomics could converge with PRS, multi-omics, and digital twin frameworks, enabling individualised simulations of drug response before therapy is initiated. This would represent a shift from reactive dose adjustment to proactive treatment optimisation, consistent with the goals of precision cardiovascular medicine.

### Implementation and clinical impact

The translation of these pharmacogenetic findings into patient care has been gradual but accelerating. Some healthcare systems and pharmacogenetics programs now genotype patients for a panel of variants (including CYP2C19, CYP2C9/VKORC1, SLCO1B1, and others), either reactively (when prescribing clopidogrel or warfarin) or proactively (by storing the data for future medications). For example, at institutions such as St. Jude and Vanderbilt University (PREDICT program), preemptive genotyping enables automated alerts in the electronic health record (EHR) when a physician orders clopidogrel for a patient with a CYP2C19*2/*2 genotype, recommending an alternative (9, 69).

Real-world evidence suggests genotype-guided therapy can improve outcomes in certain contexts. One study of acute coronary syndrome patients found that those who received genotype-tailored antiplatelet therapy (ticagrelor for CYP2C19 variant carriers) had a lower incidence of adverse cardiovascular events than those on conventional therapy (70). In another example, at the Mayo Clinic, SLCO1B1 genotyping and decision support reduced the incidence of statin discontinuation due to myalgia. These early implementation successes are encouraging, though broad adoption faces hurdles.

### Challenges to implementation

Despite robust evidence for a few gene-drug pairs, *pharmacogenomic testing is not yet standard of care* in most cardiology practices. Several factors contribute to this. First, the *level of evidence* varies: some pharmacogenetic associations are supported by multiple trials and meta-analyses (e.g., CYP2C19 and clopidogrel), whereas others show only modest effect sizes or have conflicting data. Clinicians and guideline bodies rightfully demand high levels of evidence (ideally, randomised trial data) demonstrating that using genetic info improves patient outcomes. Such trials are complex and expensive, thus only a few have been completed. Second, there are *logistical constraints*: implementing testing requires andgenotyping infrastructure for the ability to deliver results promptly. Suppose genetic testing takes 2 weeks, and an urgent therapy decision must be made sooner (as in acute coronary syndromes). In that case, it limits utility unless rapid testing or point-of-care assays are available. However, newer point-of-care genotype tests (for CYP2C19) can return results within an hour, which could be used during percutaneous coronary intervention to choose a P2Y12 inhibitor.

There are also *scientific limitations*. Pharmacogenomics traditionally focuses on one gene-one drug relationships, but drug response can be polygenic. For example, many genes influence warfarin dose aside from CYP2C9/VKORC1 (71); statin intolerance may involve a network of metabolic and immune pathways (72). A single variant test captures only part of the picture. Ongoing research into polygenic predictors of drug efficacy/toxicity could eventually enable more comprehensive risk assessments. Until then, we rely on the major known variants.

## Artificial intelligence and machine learning in cardiovascular care

The explosion of biomedical data in cardiology, from high-resolution imaging and continuous monitoring to genomic and clinical databases, has created both an opportunity and a challenge. *AI* and *ML* offer powerful tools to harness this data deluge, uncover complex patterns, and support clinical decision-making. In recent years, AI applications in cardiovascular medicine have moved from theoretical promise to tangible clinical tools, marking a pivotal shift towards data-driven personalised care (11, 12).

AI has demonstrated strong performance across multiple cardiovascular diagnostic modalities, including electrocardiography, echocardiography, cardiac MRI, CT angiography, and wearable sensor streams. Rather than treating these as separate applications, these examples illustrate a common principle: deep learning models can extract latent physiological signatures from high-dimensional data that are not readily apparent to human observers. This enables early detection of disease states, such as systolic dysfunction, arrhythmogenic substrates, and cardiometabolic derangements, using routine inputs. The clinical significance lies less in any single use case and more in the scalability of this approach, whereby inexpensive, widely available tests can be repurposed into rich phenotyping tools.

### Therapeutic decision support

While AI is not (and should not) replace clinicians, it increasingly serves as *decision support*. For example, algorithms can suggest optimal medical therapy by analyzing a patient's profile against guidelines and outcomes data, essentially as an “AI coach” to ensure no gaps in care. In complex decisions such as timing valve replacement in aortic stenosis, AI models that incorporate myriad variables may 1 day help weigh risks and benefits tailored to the individual. A burgeoning area is AI in *treatment planning* and procedure guidance. In electrophysiology, AI-driven models of cardiac electrical activity (sometimes overlapping with digital twin concepts) can inform arrhythmia ablation planning by identifying likely arrhythmic circuit locations from imaging and ECG data. There are AI prototypes that suggest catheter paths for ablation or optimal pacing sites for CRT. In interventional cardiology, robotics combined with AI image analysis may optimise stent sizing and placement or predict which lesions will benefit from intervention based on physiological and imaging features. In short, AI's pattern recognition can complement clinicians’ expertise, especially in data-intensive tasks, to tailor interventions to each patient's unique anatomy and risk profile.

### Advantages in workflow and access

A notable benefit of AI is *efficiency and standardisation*. Automated analysis can substantially reduce the time required for tasks such as imaging measurements or ECG interpretation, thereby freeing clinicians to focus on patient care. When appropriately designed and constrained for specific clinical tasks, AI systems can reduce certain forms of inter-observer variability by applying consistent feature extraction and decision rules across large volumes of data. However, many modern models, particularly generative systems, are probabilistic rather than deterministic, and their outputs can vary depending on initialisation, sampling, and prompt structure. This underscores the need for careful validation, calibration, and monitoring of clinical AI tools, especially in high-stakes decision-making contexts.

## Precision-enabled therapeutics as downstream applications of personalised frameworks

Therapeutic innovation in cardiovascular medicine should be understood not as a parallel pillar of personalised care, but as a downstream consequence of improved risk stratification, mechanistic understanding, and predictive modelling. In this framework, personalised medicine is primarily about knowing whom to treat, when to intervene, and how to tailor therapy, rather than about enumerating specific interventions. Advances in genomics, multi-omics, and artificial intelligence increasingly inform therapeutic selection, timing, and intensity, enabling a shift from uniform treatment algorithms toward individualised decision-making (52).

Accordingly, this section does not attempt to provide a comprehensive review of cardiovascular therapeutics. Instead, it highlights selected examples that illustrate how precision-enabled data frameworks may reshape future interventions. Gene editing is discussed as an extreme and future-facing case of this paradigm, alongside a brief acknowledgement of other emerging modalities.

### Mendelian cardiovascular disorders

A primary target of gene editing efforts is rare monogenic cardiovascular diseases. Conditions like familial hypercholesterolemia (FH), hypertrophic cardiomyopathy (HCM), arrhythmogenic right ventricular cardiomyopathy (ARVC), and certain channelopathies (e.g., long QT syndrome) are caused by single-gene mutations. CRISPR could theoretically fix a pathogenic mutation in a patient's cells, thereby curing the disease at its source. In *hypertrophic cardiomyopathy*, autosomal-dominant mutations in genes such as *MYH7* or *MYBPC3* lead to abnormal sarcomere proteins. Researchers have used CRISPR-Cas9 in patient-derived cells to correct an HCM mutation and normalise cellular function (73). There has even been a proof-of-concept editing of a MYBPC3 mutation in human embryos (not for clinical use, but to demonstrate the feasibility of germline correction), though germline editing raises profound ethical issues and is not being pursued clinically. For *ARVC* (often due to *PKP2* or *DSP* mutations in desmosomal proteins), gene editing in induced pluripotent stem cell (iPSC) models has recapitulated disease and tested potential gene corrections (74). Likewise, *long QT syndrome* due to SCN5A or KCNQ1 mutations has been modelled and “repaired” *in vitro*. These studies, although preclinical, highlight the precision of gene editing as a tool to address inherited cardiac conditions that currently have no cure (with only symptom management or transplantation in severe cases).

### Gene silencing for risk factors

The most groundbreaking clinical advance has been *cholesterol-lowering*. Individuals with loss-of-function mutations in the gene PCSK9 have lifelong low LDL cholesterol and markedly reduced coronary disease risk, which has inspired the development of PCSK9 inhibitors (75). Taking it a step further, biotech companies are using *in vivo* gene editing to mimic this natural protective state. In 2022 and 2023, a first-in-human trial of a base-editing therapy (VERVE-101) targeting PCSK9 was initiated in patients with heterozygous familial hypercholesterolemia, an inherited high-cholesterol disorder predisposing to premature heart disease (76). In parallel with PCSK9, ANGPTL3 has emerged as another compelling target for genome-based cardiovascular prevention. Loss-of-function variants in ANGPTL3 are associated with lifelong reductions in triglycerides and LDL cholesterol and with reduced coronary artery disease risk. Recent early-phase studies have demonstrated the feasibility of *in vivo* gene editing and silencing strategies targeting ANGPTL3, achieving durable reductions in atherogenic lipoproteins after a single intervention (77). These results extend the proof of concept that one-time genetic therapies can modulate complex lipid phenotypes and potentially address residual cardiovascular risk not fully captured by LDL-centric approaches.

Importantly, ANGPTL3 editing highlights how genetically informed target selection can guide next-generation therapeutics. Unlike traditional lipid-lowering drugs that require lifelong adherence, gene-editing strategies may enable durable or permanent modification of disease-relevant pathways. However, long-term safety, off-target effects, immune responses, and equitable access remain major concerns. As such, these therapies are best conceptualised as complements to, rather than replacements for, existing pharmacologic strategies, at least in the near term.

**Figure 2:**
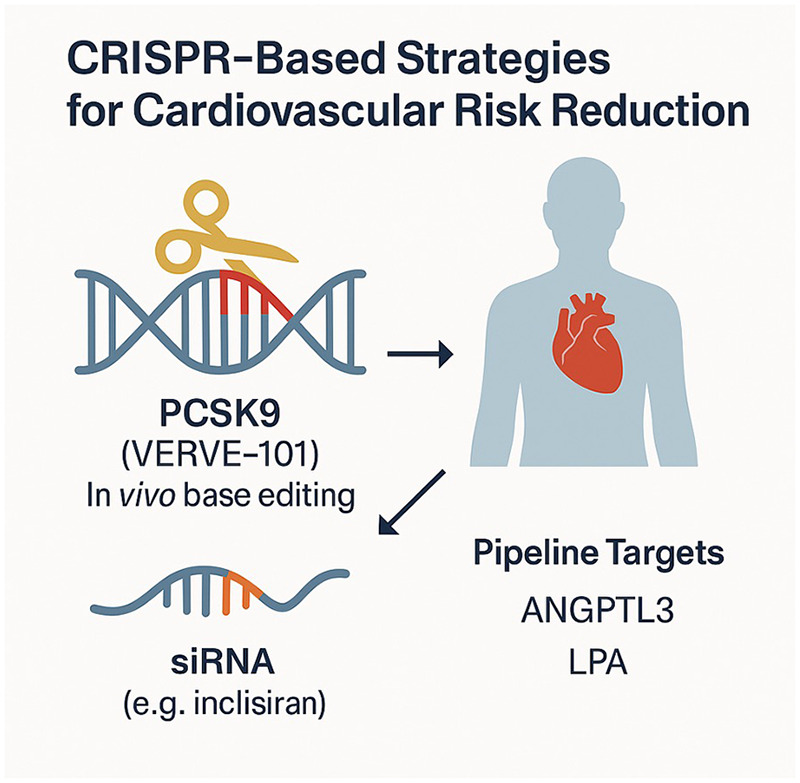
Illustration of emerging genome and transcriptome-targeting therapies aimed at lowering atherosclerotic cardiovascular risk. Base editing of the *PCSK9* gene (e.g., VERVE-101) introduces a stop codon via an *in vivo* single-nucleotide edit, permanently inactivating PCSK9 production and resulting in durable LDL cholesterol reduction. Small interfering RNA (siRNA) therapies, such as inclisiran, transiently silence *PCSK9* mRNA expression through RNA interference. Additional pipeline targets include *ANGPTL3* and *LPA*, both genetically linked to lipid metabolism and cardiovascular risk. These molecular interventions represent a shift toward one-time or infrequent therapies guided by human genetics.

Beyond gene editing, multiple therapeutic classes are already being reshaped by personalised data frameworks. RNA-based therapies, such as antisense oligonucleotides and small interfering RNAs, enable selective silencing of genetically validated targets and may be deployed based on molecular risk profiles. Biologics increasingly target inflammatory and metabolic pathways identified through proteomic and genomic analyses. Device-based therapies, including cardiac resynchronisation, neuromodulation, and ablation strategies, are being individualised through AI-guided imaging, electroanatomic mapping, and patient-specific modelling. Importantly, these interventions derive their personalised value not from novelty *per se*, but from the precision with which they are selected, timed, and titrated using integrated patient data. Looking ahead, CRISPR-based epigenome editing might be used to downregulate or upregulate specific genes without altering their sequences, offering a potentially reversible, tunable approach (Figure 2).

### Heart failure and regeneration

Beyond risk factors, gene therapy and editing are being investigated for more direct treatment of heart disease. Some preclinical studies use CRISPR to knock out genes implicated in maladaptive cardiac remodelling post-myocardial infarction (e.g., by editing specific microRNAs or myocardial receptors to mitigate fibrosis) (78). In heart failure with preserved ejection fraction (HFpEF), where effective therapies are scarce, gene expression profiles reveal upregulation of titin splicing variants and hypertrophic pathways; genome-editing tools may eventually target these mechanisms. There is also an intersection with regenerative medicine: researchers have used gene transfer (via viral vectors, not necessarily CRISPR) to induce cardiomyocyte proliferation or convert fibroblasts to cardiomyocytes *in situ*, essentially to regenerate heart muscle. While true gene-editing-based regeneration is a more distant prospect, the ability to precisely modify gene networks could facilitate tissue engineering or gene-enhanced stem cell therapies for cardiac repair.

### Current status and future directions

The field is moving swiftly. As of 2025, we have:

- Clinical trials are underway for *in vivo* base editing of PCSK9 (VERVE-101) and for an upcoming trial of ANGPTL3 (VERVE-201) in refractory hyperlipidemias.
- CRISPR-Cas9 trials for Transthyretin amyloidosis (a protein-misfolding disease affecting the heart) have shown substantial reductions in the pathogenic TTR protein after a single infusion of a CRISPR therapy. This was the first *in vivo* CRISPR trial in humans (by Intellia Therapeutics) and, although TTR amyloidosis is rare, it demonstrated the feasibility of gene editing in patients.
- *Ex vivo* editing approaches are also being explored. For example, editing a patient's cells outside the body and then reinfusing them is used for CAR-T cell therapy in cancer. In a cardiovascular context, one could imagine editing autologous stem cells or immune cells to enhance repair or reduce atherosclerosis (e.g., editing macrophages to make them resistant to cholesterol overload and then infusing them, very experimental).
- Research into gene editing for inherited cardiomyopathies is progressing toward the use of animal models. A notable success was in 2021, when base editing was used in mice to correct a dominant mutation causing HCM, preventing disease development *in vivo*. Extending that to larger animals and, eventually, to patients is a goal, albeit with many hurdles.

Beyond gene editing, multiple therapeutic classes are already being reshaped by personalised data frameworks. RNA-based therapies, such as antisense oligonucleotides and small interfering RNAs, enable selective silencing of genetically validated targets and may be deployed based on molecular risk profiles. Biologics increasingly target inflammatory and metabolic pathways identified through proteomic and genomic analyses. Device-based therapies, including cardiac resynchronisation, neuromodulation, and ablation strategies, are being individualised through AI-guided imaging, electroanatomic mapping, and patient-specific modelling. Importantly, these interventions derive their personalised value not from novelty *per se*, but from the precision with which they are selected, timed, and titrated using integrated patient data.

In the long run, *genome editing and gene therapy could fundamentally alter the management of cardiovascular disease*. We might shift from lifelong pharmacotherapy to “one-and-done” treatments for certain conditions. Imagine a middle-aged patient with high Lp(a) and early coronary disease; instead of decades of aphereses or new drugs, a single gene-silencing injection could permanently reduce Lp(a) production. Or a young individual with hypertrophic cardiomyopathy mutation, an infusion of gene editors could silence the mutant gene and halt disease progression, avoiding defibrillator shocks or transplant. While this sounds futuristic, the early building blocks are here.

Of course, many cardiovascular diseases are complex and polygenic, not amenable to a single gene fix. However, even there, gene editing might contribute indirectly by creating better models to study polygenic disease or by modifying risk in combination with other therapies. For common polygenic diseases, editing multiple genes is impractical; however, editing a regulatory gene or master switch that influences a network (for example, a transcription factor that drives atherosclerosis pathways) could yield benefits, though identifying such targets is challenging.

## Digital twin modelling in cardiology: virtual patients for personalized care

Imagine having a virtual replica of a patient, a *digital twin*, that can model the patient's cardiovascular system, predict future disease events, and simulate responses to various treatments. What sounds like science fiction is becoming a tangible goal in precision medicine. A digital twin is a computational model of a specific patient, constructed from the individual's data (genomic, clinical, imaging, sensor data, etc.) and updated continuously or periodically with new data, mirroring the patient's current state (79). In cardiovascular medicine, digital twin concepts are explored to personalise risk prediction and therapy planning, leveraging advanced modelling and AI.

### Principles of the digital twin

A cardiovascular digital twin framework entails integrating multiple layers of information about a person into a cohesive model. This can include:

- *Anatomical data*: e.g., a 3D model of the patient's heart and vasculature derived from imaging (MRI/CT) or ultrasounds.
- *Physiological data*: vital signs, hemodynamic measurements (like blood pressure, blood flow velocities), and lab values.
- *Pathophysiological behavior*: models of electrophysiology (heart rhythm), muscle contraction, fluid dynamics of blood flow, etc., often based on biophysical equations.
- *Personal data streams*: wearable device data (heart rate trends, activity, sleep), environmental exposures.
- *Genomic and molecular profiles*: any patient-specific biomarkers or genetic predispositions that might affect disease processes.

These elements are combined using mathematical models and AI algorithms to create an individualised simulation. For example, a digital twin might incorporate a multi-scale heart model (from ion channels in cells to tissue-level electrophysiology to whole-heart motion and circulation).

Crucially, a digital twin is not static; it can assimilate new data (e.g., a patient gaining weight, blood pressure increasing, or receiving a new laboratory result) and update its predictions. It's akin to a “cyber-physical system” in which data flows between the patient and the twin in real-time or near-real-time. Achieving this level of synchronisation is challenging, but the vision is that the twin could forecast outcomes such as when heart failure decompensation is likely, or how the coronary plaque in its virtual arteries will progress under different scenarios (e.g., continue smoking vs. quit smoking).

### Current progress and applications

The digital twin concept is still emerging in healthcare, but some initial applications in cardiology demonstrate its potential:

- *Cardiac Electrophysiology Twins*: Researchers led by Natalia Trayanova at Johns Hopkins have created patient-specific heart models (virtual hearts) to guide therapy for arrhythmias. By inputting a patient's cardiac MRI scar data and electrical mapping data, they generate a digital twin heart that can be stimulated *in silico* to identify arrhythmia circuits. In ventricular tachycardia (VT) ablation, such models have been used to predict which scar regions are critical for VT maintenance and should be targeted (12). A recent retrospective study showed that digital twin simulations could noninvasively predict VT ablation sites that matched those identified clinically (80). This approach is being tested prospectively: before an ablation, simulate to plan it, potentially reducing procedure time and improving success. Similarly, in AF, digital twin models of the atria (incorporating fibrosis data from imaging) are being studied to predict ablation outcomes and to test drugs (a virtual “trial” of antiarrhythmic drug effects on that patient's atria).
- *Hemodynamic Twins*: In diseases like aortic stenosis or heart failure, patient-specific computational models of the heart and circulation can predict hemodynamic consequences of interventions. For example, the TwinCardio framework has been proposed to detect and monitor cardiovascular disease by creating a digital replica that can test scenarios (81). One could simulate how a particular patient's cardiac output and pressures would change after valve replacement or in response to a specific drug dose. This might aid decision-making, e.g., in borderline aortic stenosis cases where it's unclear whether valve replacement will improve function; a digital twin could model the outcome.
- *Virtual Trials and Risk Prediction*: On the prevention side, digital twins could be used to run “virtual trials” for an individual. If we have a validated model for, say, coronary heart disease progression that incorporates risk factors, one could simulate the patient under a given intervention (e.g., starting a high-intensity statin or achieving a 10 mmHg BP reduction) and estimate how the patient's risk curve changes. One recent concept is combining digital twins with general AI to accelerate precision prevention (82). For instance, a twin might incorporate personal risk factors, and then an AI can project individualised risk trajectories under different lifestyle or treatment modifications. An early example is a mechanistic model simulating the evolution of risk factors (weight, diabetes, blood pressure) to predict long-term outcomes (83). Although rudimentary, it illustrates how a twin might predict disease progression and enable proactive intervention.
- *Device Testing*: Before implanting a device or performing a surgery, a digital twin could be used to test-fit and optimise. In structural heart interventions, having a patient's heart model enables virtual deployment of a TAVR (transcatheter aortic valve) or a left atrial appendage occlusion device to assess fit and hemodynamic effects, thereby supporting sizing and approach. There are reports of using 3D-printed models for this; digital twins would do it computationally.
- *Personalised Patient Engagement*: Another approach is to use the digital twin as a patient education and engagement tool. If you could show patients a simulation of their own heart and how, for example, their blood pressure is making it work harder, or how quitting smoking would slow their plaque growth, it might motivate adherence to therapies. It personalizes the consequences of risk factors beyond generic statistics.

Digital twins should be viewed as complementary to, rather than replacements for, *in vitro* patient-specific modelling approaches such as induced pluripotent stem cell (iPSC)-derived cardiomyocytes, engineered heart tissues, and cardiac organoids. While digital twins aim to simulate organ-level and system-level physiology by integrating multi-source patient data, *in vitro* platforms provide cellular- and tissue-level experimental systems that capture patient-specific genetic and epigenetic contexts under controlled conditions. These models allow direct observation of electrophysiological properties, contractility, calcium handling, and drug responses in living human-derived tissue, offering a level of mechanistic granularity that current *in silico* models cannot fully replicate (84).

However, *in vitro* systems also face important limitations. iPSC-derived cardiomyocytes often resemble fetal rather than adult phenotypes, lack native multicellular architecture, and do not fully recapitulate the biomechanical, neurohormonal, and vascular influences present *in vivo*. Moreover, they remain resource-intensive, time-consuming to generate, and impractical for routine clinical use. By contrast, digital twins can operate at the whole-organism scale, integrate longitudinal clinical and wearable data, and simulate multiple therapeutic scenarios in real time, making them more suitable for clinical decision support (85).

Future precision cardiology frameworks may integrate both paradigms into hybrid models, in which patient-derived tissues inform parameterisation and validation of digital twins, while *in silico* simulations guide experimental testing of therapies. Such bidirectional coupling could enable a new class of personalised models that combine biological fidelity with clinical scalability, bridging mechanistic insight and real-world decision-making.

### Challenges

Building a true digital twin in cardiology is extraordinarily complex. The cardiovascular system is governed by multiscale physics (electrical, mechanical, fluid dynamics) and influenced by neurohormonal and metabolic factors. Creating a comprehensive model of a single patient that accurately captures all relevant processes is a significant challenge. Key obstacles include:

- *Data integration*: Twins require pooling data from disparate sources/formats (e.g., DICOM imaging, continuous wearables, laboratory values) into a single model. Ensuring interoperability and standardisation is not trivial (86).
- *Model validity*: The underlying models must be physiologically sound and personalised via patient data. Population-based models need to be adjusted to individual parameter values. If a model is too generic, it won't make accurate predictions for a given person. Conversely, making it individual might require more data than we usually have (e.g., tissue properties of a specific heart).
- *Real-time update*: The ideal twin updates with new data. But practical issues arise: how to feed data continuously (for example, a blood pressure reading every minute from a smartwatch, do we plug that in continuously?), and how to recalibrate the model on the fly. It requires robust algorithms that can integrate data while remaining stable.
- *Computational load*: Detailed simulations (e.g., finite-element models of heart mechanics or fluid-dynamics simulations of blood flow) are computationally intensive. Running these in real time is challenging. Approaches to mitigate this include using reduced-order models or AI surrogates (train an AI to emulate the physics model, which runs faster).
- *Validation*: A digital twin's predictions must be validated against reality. For example, if the twin predicts a certain risk of heart failure exacerbation in the next month, or a certain blood pressure result after a medication change, those need to be checked in practice. We need to establish trust that the model reflects accurately accurate reality. This likely means running studies where care guided by digital twin vs. standard care are compared.
- *User interface*: For a clinician to use a digital twin, the output needs to be presented understandably. Whether it's a dashboard of risk projections or a simulation video, it must highlight actionable information. Too much complexity could overwhelm users.
- *Ethical concerns*: Since digital twins rely on comprehensive data, privacy is paramount. A twin contains essentially all known information about a person's health, a treasure trove that must be protected from misuse. Also, decisions guided by a twin raise questions of liability (if the twin was wrong) and transparency (patients should know if a simulation influenced their treatment).

### Future perspectives

Despite challenges, enthusiasm for digital twins in healthcare is high because of the potential for *truly individualised prediction and treatment*. Research and development are accelerating:

- The 2022 review by Coorey et al. noted that digital twin research in CVD is growing globally with academic and commercial stakeholders, and that most efforts to date are focused on numerical simulation models that are precursors to fully integrated real-time twins. They identified multiple companies investing in health digital twins and several patents filed for cardiovascular simulation methods (86).
- By 2025, we are seeing early integration of AI to make twin models more intelligent and adaptive. For instance, AI could learn from population data to fill in gaps in a person's twin model (like estimating an unknown parameter from known ones), essentially *hybrid modeling* combining physics-based and data-driven approaches (82).
- One can expect incremental progress: first, digital twin-like decision support may emerge for specific use cases (e.g., arrhythmia ablation planning, as mentioned, or a twin-based risk calculator for ICU settings to predict shock or arrhythmias). Over time, as data capture improves (with ubiquitous sensors and better EHR integration), more continuous and holistic twins become feasible.
- In the longer term, if validated, digital twins could revolutionise clinical trials. Instead of randomising thousands of patients, one could simulate multiple virtual cohorts using twins to test hypotheses or assess drug effects before enrolling in actual trials. This is speculative, but researchers have proposed “*in silico* trials” to complement traditional trials.

Importantly, digital twin technology may reinforce patient engagement: patients could interact with their own digital model through apps, viewing projections and managing their health as a strategy game (with appropriate guidance). This remains conceptual but not impossible as user interfaces improve.

**Figure 3:**
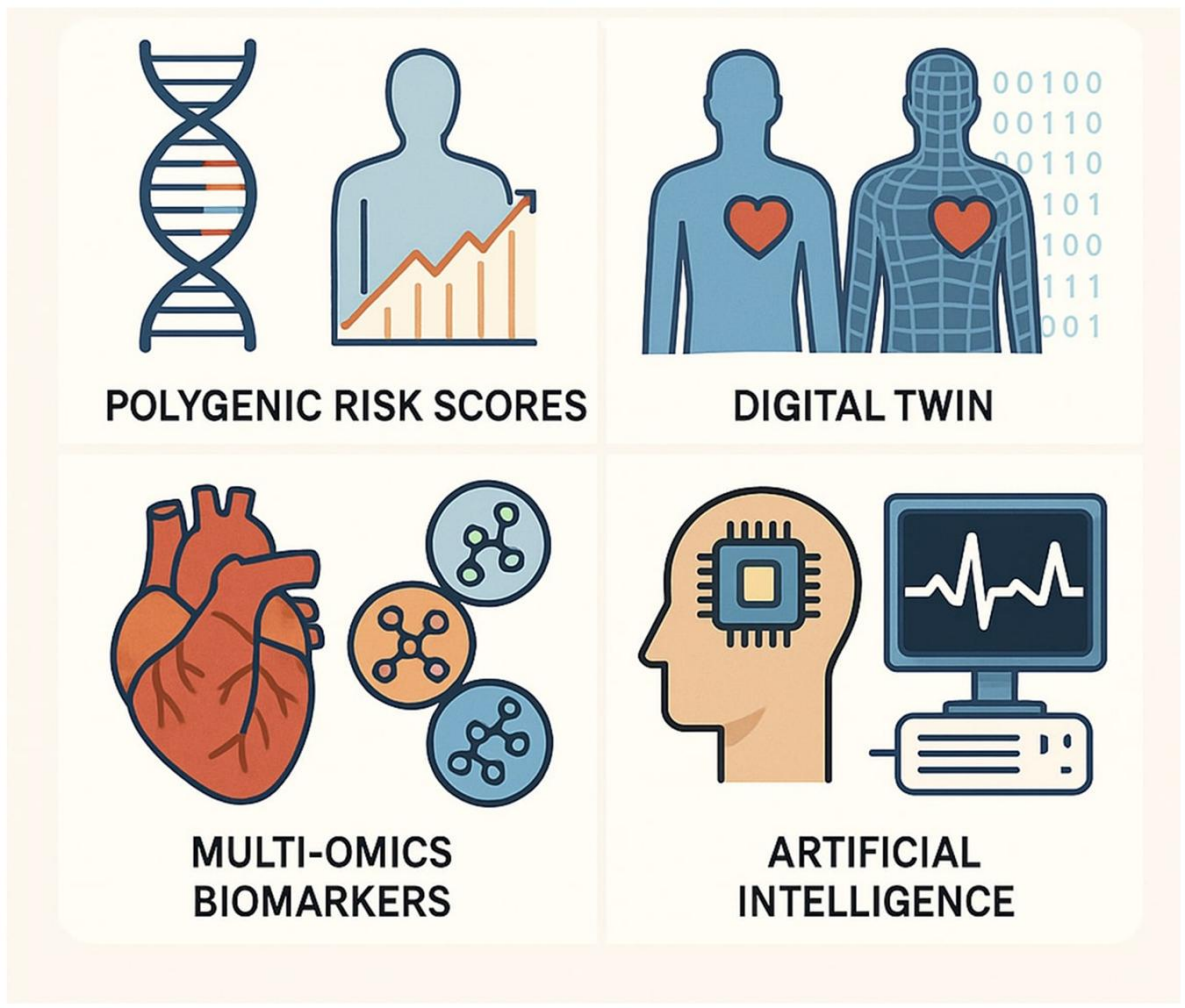
An illustration showing the key pillars of personalised cardiovascular medicine. Top left: *Polygenic Risk Scores* integrate genetic variants to estimate inherited risk for cardiovascular diseases. Top right: *Digital Twin* technology creates a dynamic virtual replica of an individual, enabling simulation of disease progression and treatment outcomes. Bottom left: *Multi-Omics Biomarkers* combine data from genomics, proteomics, and metabolomics to enhance diagnosis and risk stratification. Bottom right: *Artificial Intelligence* processes complex clinical and physiological data to support precision diagnostics, prognostication, and therapeutic decision-making. Together, these innovations underpin the transition toward proactive, individualised cardiovascular care.

**Figure 4:**
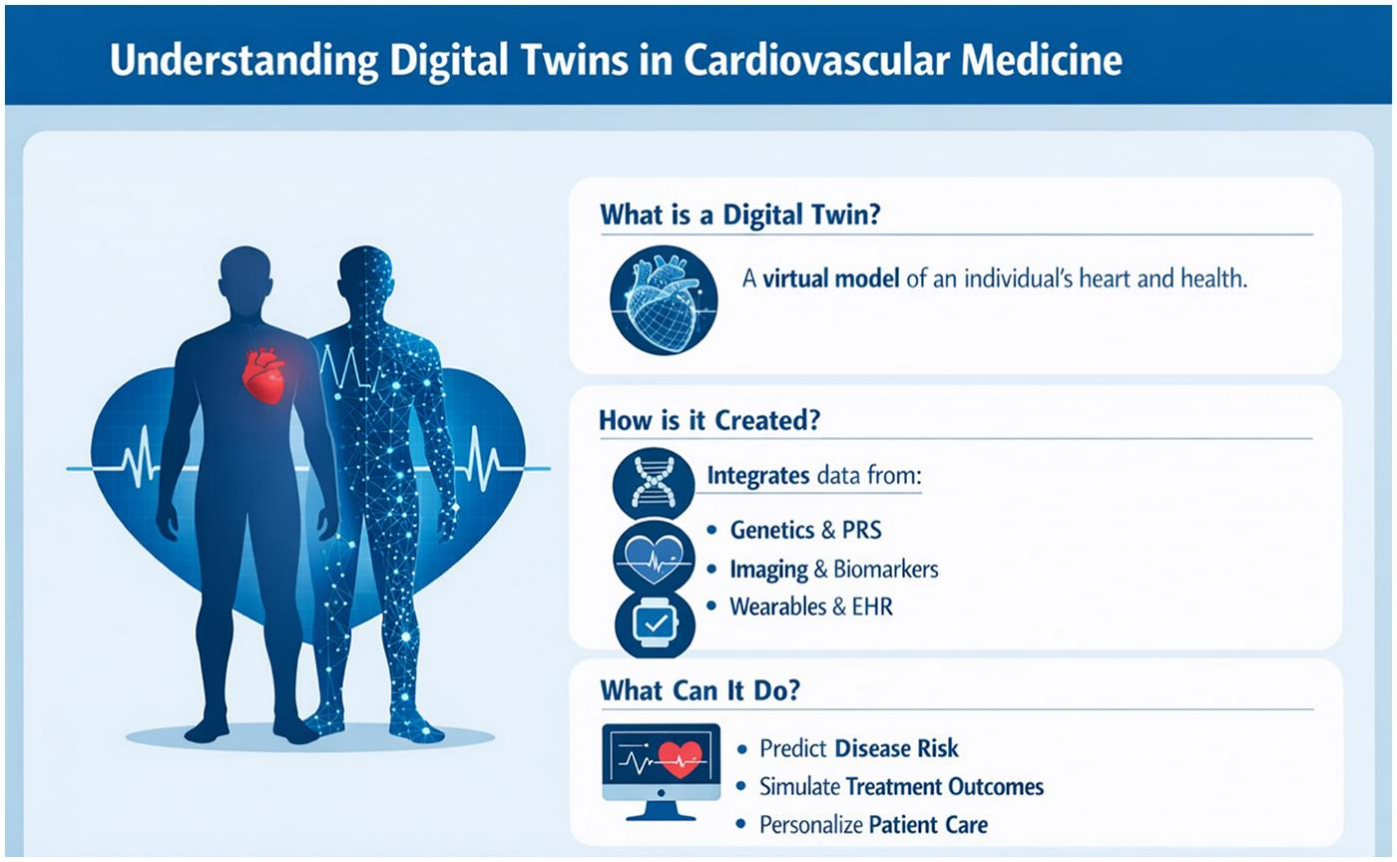
Digital twins in personalised cardiovascular medicine. Digital twins are patient-specific virtual replicas that integrate multi-source data, including genetics, polygenic risk scores, imaging, biomarkers, electronic health records, and wearable sensor data. These data streams are combined using biophysical modeling and artificial intelligence to simulate cardiovascular structure, function, and disease trajectories *in silico*. Digital twins can be used to forecast risk, simulate treatment responses, and support individualized clinical decision-making before interventions are applied in real patients. This approach enables proactive, model-informed care rather than reactive management.

Table 1 summarises key pillars in personalised cardiovascular medicine, highlighting their current clinical utility, limitations, and prospects. Also, Figure 3 demonstrates the key pillars of personalised cardiovascular medicine. Figure 4 provides a visual overview of the digital twin concept, illustrating how multi-source patient data are integrated into a virtual model to support prediction, simulation, and personalised care.

**Table 1:** Core pillars of personalised cardiovascular medicine: clinical role, readiness, and implementation priorities.

Pillar	What it enables in practice	Current readiness	Key limitation	Next step to clinical adoption
Polygenic risk scores	Early-life risk stratification and risk-enhancer for preventive decisions	Moderate	Reduced portability across ancestries and unclear action thresholds	Multi-ancestry calibration and trials testing PRS-guided prevention pathways
Multi-omics biomarkers	Mechanistic phenotyping, refined risk stratification, target discovery	Low to moderate	High cost, assay variability, uncertain actionability	External validation and development of clinically actionable panels
Pharmacogenomics	Drug selection and dosing, fewer adverse events	Moderate	Turnaround time and inconsistent guideline uptake	Pre-emptive panel testing with EHR decision support
AI and machine learning	Automated interpretation and scalable prediction from imaging, ECG, EHR, wearables	Moderate	Bias, lack of explainability, limited prospective validation	Prospective multi-site validation and workflow-integrated deployment
Gene editing and gene silencing	Durable LDL lowering and potential one-time disease modification	Early	Long-term safety, delivery challenges, cost, equity	Large trials with long-term safety monitoring
Single-cell genomics	Cell-type specific mechanism discovery and target prioritisation	Early	Tissue access, complexity, lack of clinical pathways	Translation into biomarkers and minimally invasive assays
Digital twin models	Patient-specific simulation for planning and forecasting	Early	Data integration, compute burden, limited validation	Use-case specific twins with prospective outcome evaluation

## Conclusion

Personalised cardiovascular medicine is advancing rapidly, driven by innovations in genetics, omics, data science, and bioengineering. This review traced the evolution from PRS to digital twins, illustrating how each domain supports individualised CVD care. PRS integrate millions of variants to stratify risk and enable early intervention. Multi-omics biomarkers reflect dynamic molecular states, refining diagnosis and revealing novel therapeutic targets. Pharmacogenomics addresses interindividual variability in drug responses, thereby improving safety and efficacy. AI and ML extract patterns from complex clinical and sensor data, enhancing diagnostic precision and risk prediction. Gene editing and related therapies offer the potential to correct disease at its source, while single-cell approaches map disease at unparalleled resolution. Digital twins unify these inputs into continuously adaptive virtual models to guide care.

Across all domains, common translational challenges emerge. These include the need for prospective validation, harmonised data standards, equitable representation in training cohorts, regulatory clarity, and integration into real-world clinical workflows. Rather than addressing these issues independently within each technological silo, future progress in personalised cardiology will depend on unified implementation frameworks that emphasise interoperability, continuous learning, and patient-centred governance. This reinforces the central thesis of this review: that the transformative potential of precision tools lies not in their individual sophistication, but in their coordinated deployment within adaptive, model-informed care systems. The next phase of personalised cardiovascular medicine will be defined less by technological novelty than by implementation success. Priority areas include generating prospective, outcome-driven evidence; developing interoperable, workflow-integrated decision-support systems; and establishing governance frameworks that ensure transparency, safety, and equitable access. Without deliberate investment in these domains, precision tools risk remaining academically impressive but clinically peripheral. A shift toward implementation science, pragmatic trials, and inclusive data infrastructure will be essential to translate molecular and computational advances into durable population-level benefits. If responsibly deployed, personalised cardiology has the potential to transform not only how we predict and treat disease, but how we design learning healthcare systems that continuously adapt to individual and societal needs.
